# Controlled Direct-Vision Intramuscular Gluteal Augmentation Using Macro-Bipolar Energy: A Retrospective Single-Center Study

**DOI:** 10.7759/cureus.103777

**Published:** 2026-02-17

**Authors:** Hector Alvarez-Trejo, Carina M Álvarez-Dávalos, Erick M Hernández-Mancillas, Quitzia L Torres-Salazar

**Affiliations:** 1 Surgery, Instituto de Seguridad y Servicios Sociales de los Trabajadores del Estado, Guadalajara, MEX; 2 Surgery, Centro Universitario de Ciencias de la Salud, Universidad de Guadalajara, Guadalajara, MEX; 3 Surgery, Universidad Autónoma de Durango, Durango, MEX; 4 Biomedical Sciences, Universidad Juárez del Estado de Durango, Durango, MEX

**Keywords:** buttocks, macro-bipolar energy, operative, silicone implants, surgical procedures

## Abstract

Introduction

Intramuscular gluteal augmentation with silicone implants is an established surgical approach aimed at improving implant coverage and contour stability. Conventional intramuscular dissection techniques involve limited visualization, which may affect pocket definition and intraoperative control. Direct-vision dissection represents a technical refinement designed to allow more controlled intramuscular pocket creation. The objective of this study was to describe a controlled direct-vision technique for intramuscular gluteal augmentation using macro-bipolar energy and to evaluate its clinical and aesthetic outcomes.

Methods

A single-center retrospective observational study was conducted including 17 consecutive female patients who underwent primary intramuscular gluteal augmentation with round silicone implants. All patients were considered unsuitable candidates for gluteal fat grafting and underwent implant placement entirely within the gluteus maximus muscle through direct-vision dissection of the FROD space assisted by macro-bipolar energy. Demographic data, operative characteristics, and postoperative outcomes were analyzed using descriptive statistics. Aesthetic outcomes were assessed through a combined approach including surgeon clinical evaluation, serial photographic comparison, and patient-reported satisfaction. Functional outcomes, particularly pain or discomfort while sitting, were actively assessed at each postoperative visit.

Results

The median age was 30 years (25-34), and the median body mass index was 28 kg/m² (27-29). The median operative time was 60 minutes (52-60), and implant volumes ranged from 360 to 415 cc. The mean postoperative follow-up was 24 months. Fifteen patients (88.2%) had an uncomplicated postoperative course. One late seroma and one postoperative hematoma were observed. No cases of infection, implant malposition, palpability, herniation, sciatic nerve injury, or revision surgery were documented. Adequate gluteal projection and bilateral symmetry were achieved in all patients based on clinical evaluation, photographic comparison, and patient-reported satisfaction. No functional limitations or persistent pain while sitting were reported during follow-up.

Conclusions

Intramuscular gluteal augmentation performed using a direct-vision dissection technique assisted by macro-bipolar energy appears to be a safe and reproducible approach in this retrospective cohort. The technique was associated with a low complication rate, efficient operative time, and consistent aesthetic outcomes. Further prospective studies are warranted to confirm these findings and to assess long-term results.

## Introduction

Gluteal augmentation has become one of the fastest-growing procedures in aesthetic body contouring worldwide. Silicone implants remain a valuable option, particularly in patients who are not suitable candidates for autologous fat grafting due to low body mass index or limited donor fat availability. In this context, intramuscular placement of gluteal implants has been widely adopted, as it provides improved implant coverage, enhanced stability, and a lower risk of palpability or malposition when compared with subfascial techniques [1]. This technical refinement is particularly relevant in light of recent safety concerns surrounding gluteal augmentation procedures, underscoring the importance of controlled anatomical dissection and strict respect for intramuscular planes to minimize preventable complications.

Despite these advantages, conventional intramuscular gluteal augmentation relies on blunt, blind dissection of the implant pocket within the gluteus maximus muscle, typically in the intramuscular space described by Rodríguez-García et al. as the FROD space [2]. This approach, although effective, may be associated with imprecise pocket design, increased tissue trauma, limited hemostatic control, and greater variability in surgical outcomes. Such factors can contribute to longer operative times and may increase the risk of complications such as hematoma, seroma, or implant malposition [3].

In recent years, growing attention has been directed toward refining surgical techniques that allow for greater anatomical precision, improved intraoperative control, and reduced tissue aggression. Direct-vision dissection techniques, combined with advanced energy-based devices, offer the potential to overcome some of the inherent limitations of blind intramuscular dissection. Macro-bipolar energy, in particular, enables controlled tissue separation with simultaneous hemostasis, allowing the accurate identification of intramuscular planes while minimizing bleeding and collateral muscle trauma [4].

The purpose of this case series is to describe a refined surgical technique for intramuscular gluteal augmentation based on direct-vision dissection of the FROD space using macro-bipolar energy and to evaluate its feasibility, safety, and aesthetic outcomes in a consecutive series of patients. By presenting our experience, we aim to contribute a reproducible and controlled technical alternative to conventional intramuscular gluteal implant placement.

## Materials and methods

This manuscript corresponds to a single-center retrospective observational study of consecutive patients who underwent intramuscular gluteal augmentation with silicone implants using a refined surgical technique based on direct-vision dissection of the FROD space assisted by macro-bipolar energy. A total of 17 female patients were included. All patients presented with platypygia and sought surgical augmentation to achieve increased gluteal volume and projection. Eligible patients had a body mass index lower than 29 kg/m², were between 21 and 35 years of age, and were not considered suitable candidates for gluteal fat grafting due to limited adipose tissue availability. Patients with previous gluteal implant surgery, combined augmentation techniques, or incomplete clinical documentation were not included.

All procedures were performed at a single private surgical center, Hospital de Padua, located in Zapopan, Jalisco, Mexico, by the same senior surgeon as part of standard clinical practice. The study retrospectively reviewed patients who underwent intramuscular gluteal augmentation between January 1 and December 31, 2025. Given the retrospective, observational, and non-interventional nature of the study, formal approval by an ethics committee was not required in accordance with local regulations. Written informed consent was obtained from all patients prior to surgery, including explicit authorization for the use and publication of anonymized clinical data and photographic images. This study was reported in accordance with the Strengthening the Reporting of Observational Studies in Epidemiology (STROBE) guidelines [5].

**Figure 1:**
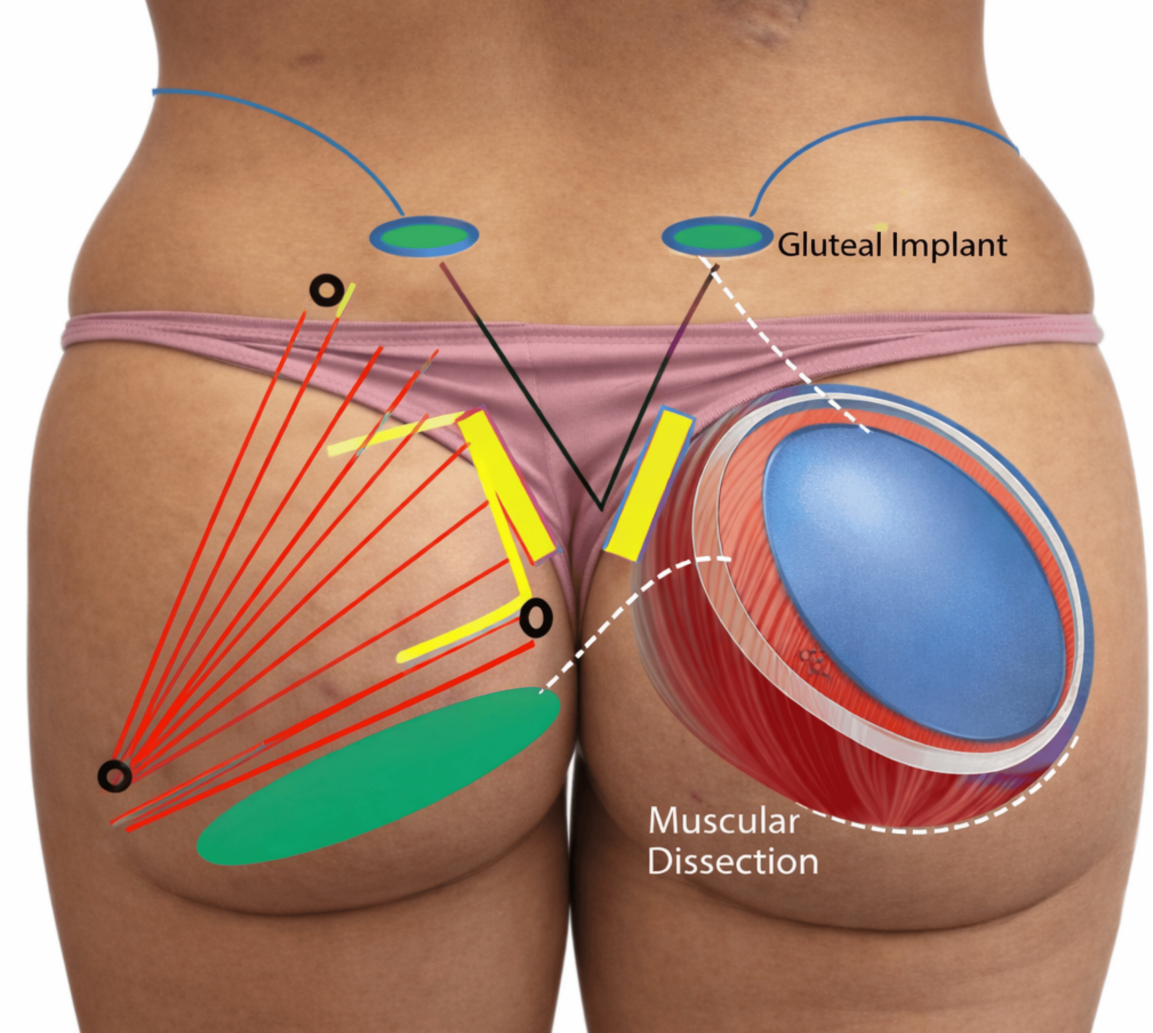
Anatomical landmarks and surgical planning for intramuscular gluteal augmentation Black dots indicate the superior, medial, and lateral limits of the gluteus maximus muscle. Red lines represent the orientation of the gluteus maximus muscle fibers. Yellow markings indicate the planned skin incisions. The blue area delineates the intended intramuscular implant pocket. The green zone corresponds to a safety area intentionally preserved free of dissection and implant placement. This figure is an original illustration created by the authors.

All surgeries were performed under general anesthesia with the patient positioned prone. Preoperative markings were carried out with the patient standing, delineating the superior, medial, and lateral borders of the gluteus maximus muscle (Figure 1).

**Figure 2:**
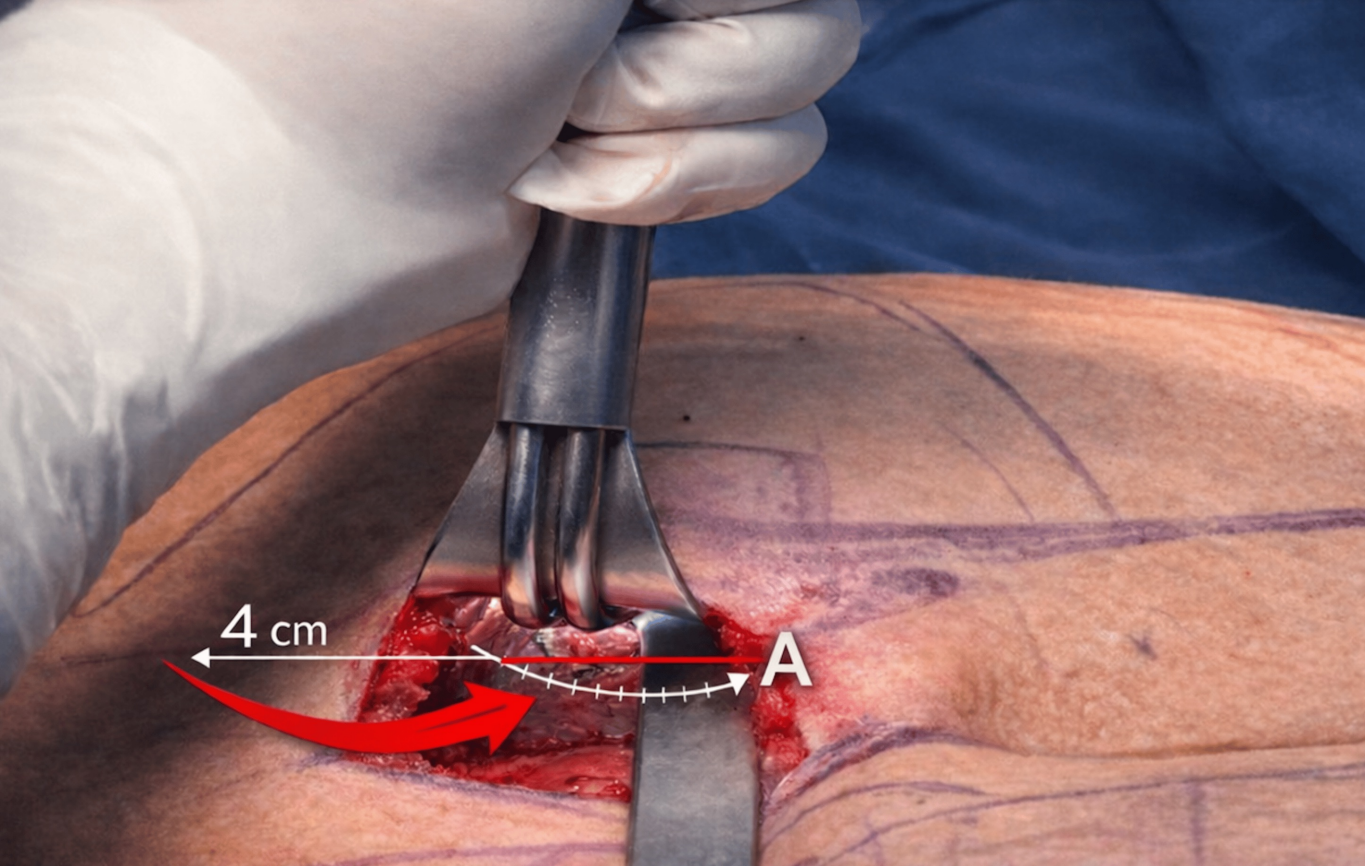
Initial subcutaneous dissection and macro-bipolar dissection of the FROD space under direct vision The red arrow indicates the approximately 4 cm subcutaneous dissection plane. The letter A identifies the fascia and muscle mass of the gluteus maximus muscle, which represent critical anatomical structures that must be preserved to allow a tension-free, three-layer closure. This figure is an original illustration created by the authors.

Two semicircular medial incisions of approximately 5 cm were planned over the upper gluteal fold, separated by a skin bridge greater than 1 cm incorporating the sacrocutaneous ligament. After skin incision, an initial subcutaneous dissection of approximately 4 cm was performed to expose the fascia and muscle mass of the gluteus maximus, preserving the structures required for a tension-free layered closure (Figure 2).

**Figure 3:**
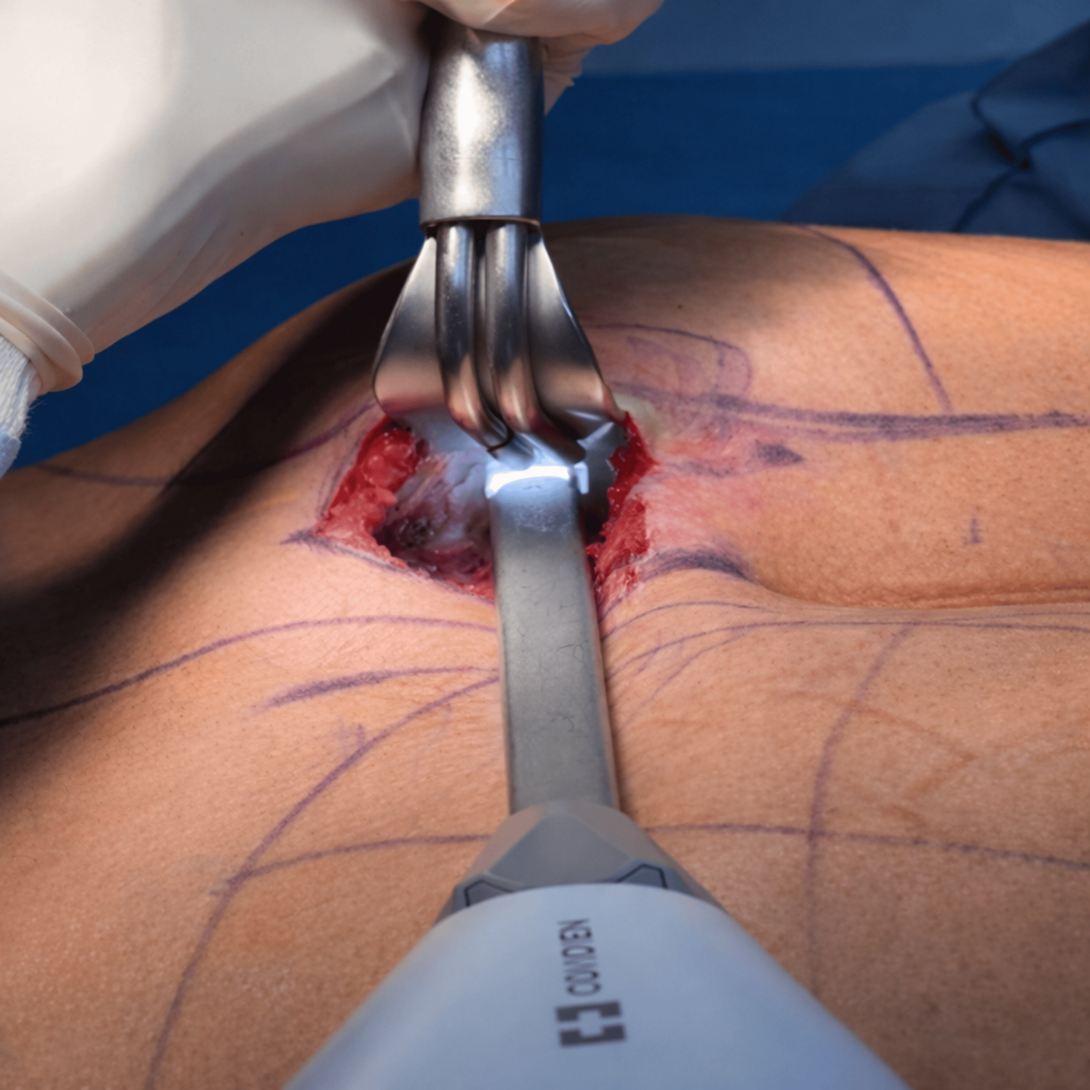
Direct-vision dissection of the FROD space using macro-bipolar energy Controlled separation of gluteus maximus muscle fibers is performed under direct visualization, allowing effective hemostasis and precise intramuscular pocket formation. The surgical field appears clean and well-defined, with minimal bleeding observed during dissection. Complete intramuscular pocket creation was achieved in approximately 15 minutes.

Under direct visualization, controlled intramuscular dissection was performed within the FROD space of the gluteus maximus muscle using macro-bipolar energy (Figure 3). Macro-bipolar dissection was performed using a LigaSure™ device set at 60% power, with a standardized activation time of approximately three seconds per application, using an Impact™ forceps for open surgery in all cases. This approach allowed the precise separation of muscle fibers with simultaneous hemostasis, resulting in a clean and well-defined intramuscular pocket with minimal bleeding. The intramuscular pocket measured approximately 10 cm in diameter and was created in an average time of 15 minutes (Figure 3).

**Figure 4:**
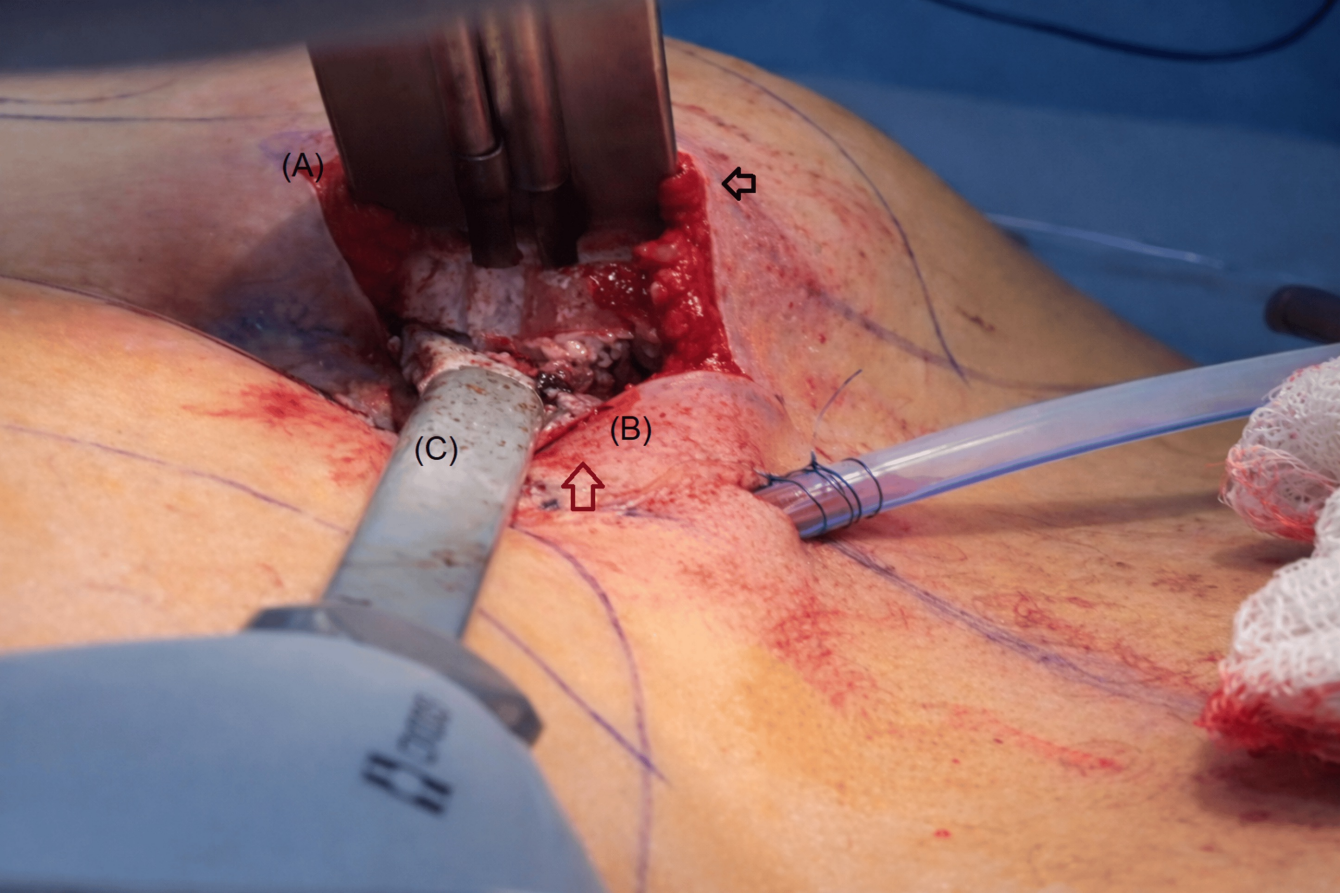
Verification of intramuscular pocket boundaries under direct visualization Direct separation using a lighted Tebbetts retractor allows clear identification of the roof and floor of the intramuscular pocket. Letter A marks the muscle pocket ceiling, and letter B identifies the muscle pocket floor, both covered by preserved gluteus maximus muscle layers measuring approximately 1-2 cm in thickness. Letter C indicates the macro-bipolar dissector used for controlled intramuscular separation. The black arrow highlights the roof of the pocket with preserved muscle fibers within the FROD space, while the red arrow identifies the pocket floor with visible muscle thickness, confirming complete intramuscular implant coverage. This figure is an original illustration created by the authors.

Particular care was taken to preserve the fibrous tissue of the ischial tuberosity, maintaining this area free of implant placement. Direct separation using a lighted Tebbetts retractor was employed to confirm the integrity of the roof and floor of the pocket, ensuring a muscle thickness of at least 1-2 cm at both levels to guarantee complete intramuscular implant placement and reduce the risk of palpability, herniation, or chronic pain (Figure 4).

**Figure 5:**
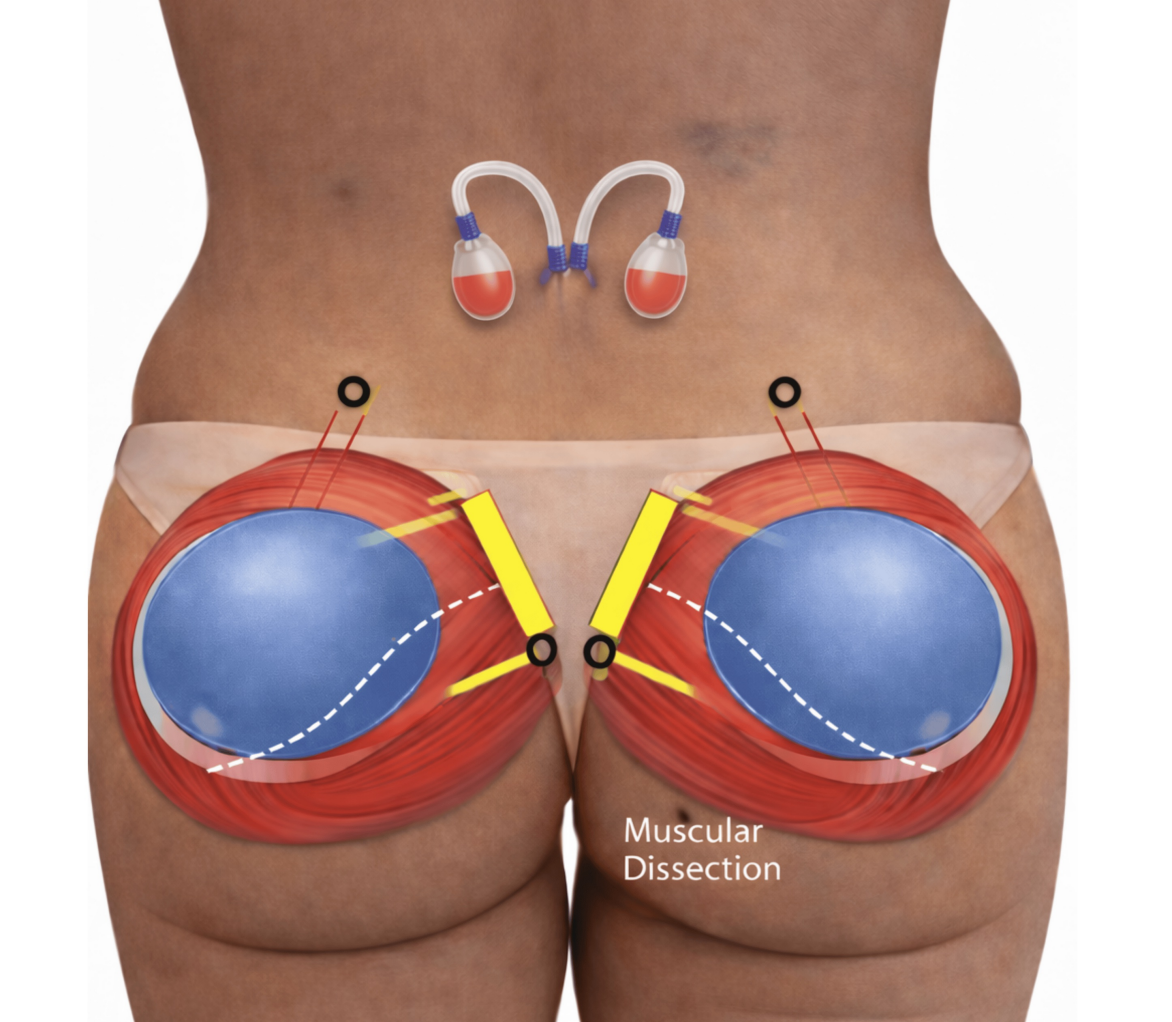
Schematic representation of intramuscular gluteal implant placement The illustration depicts bilateral intramuscular positioning of round silicone implants within the gluteus maximus muscle following controlled dissection of the FROD space. Yellow markings indicate the planned surgical access and direction of intramuscular dissection. The blue region delineates the intended intramuscular implant pocket within the gluteus maximus muscle. White lines represent the orientation of muscle fibers and the limits of the intramuscular plane. Black circles identify key anatomical reference points used to guide pocket creation and implant positioning. The schematic emphasizes complete muscular coverage of the implants and preservation of surrounding anatomical safety zones. This figure is an original illustration created by the authors.

Round silicone gluteal implants with volumes ranging from 360 to 500 cm³ were placed entirely within the gluteus maximus muscle following controlled intramuscular dissection. Figure 5 provides a schematic representation of the standardized intramuscular implant positioning and pocket orientation, illustrating the intended relationship between the implant, muscle fibers, and preserved safety zones.

**Figure 6:**
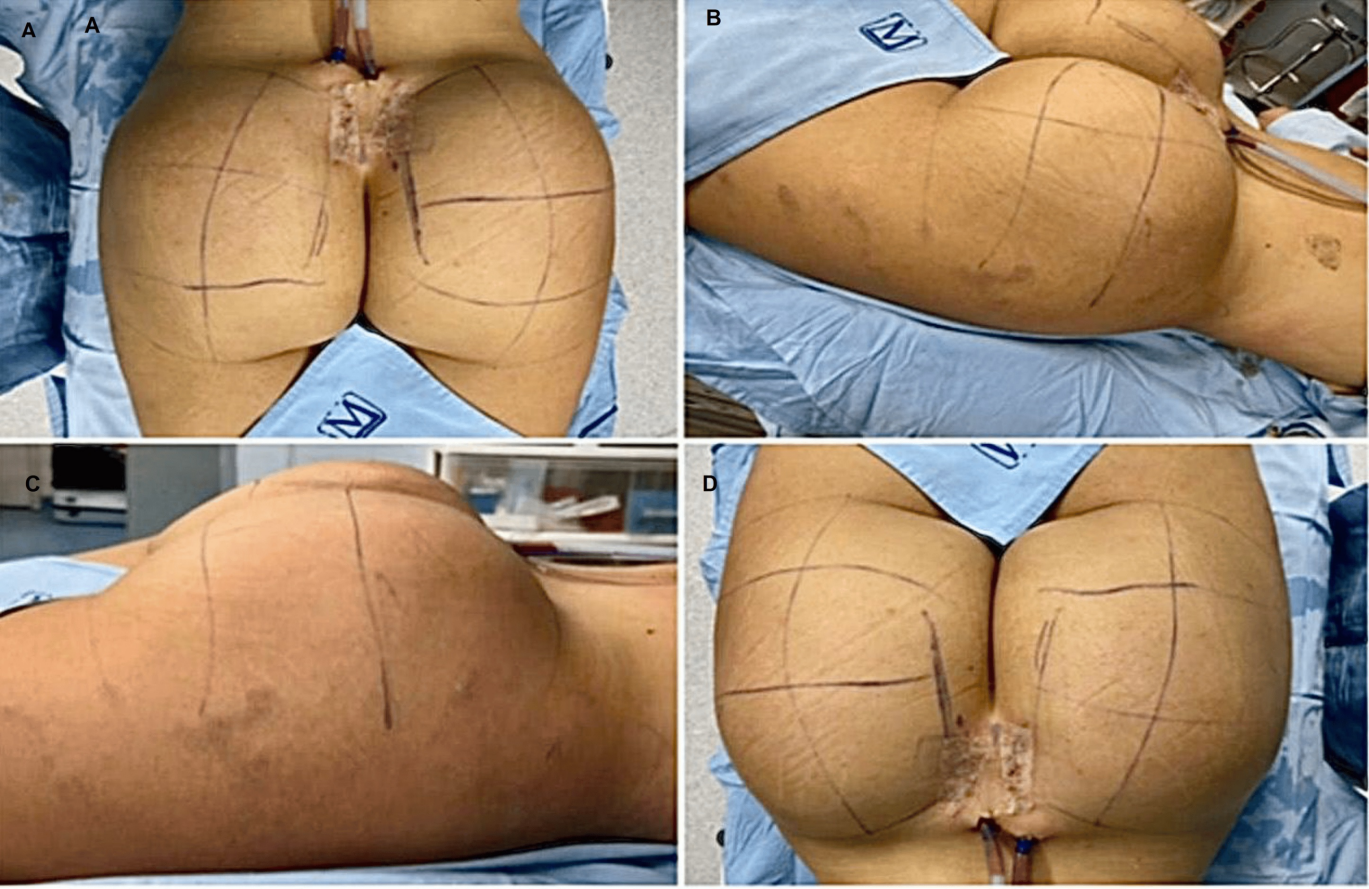
Immediate postoperative outcome (A) Posterior view showing bilateral gluteal augmentation with symmetric projection and preserved intergluteal contour. (B) Oblique lateral view demonstrating adequate gluteal projection and smooth contour transition. (C) Lateral view highlighting uniform volume distribution and absence of surface irregularities. (D) Inferior-posterior view illustrating central scar position with appropriate wound closure and maintained bilateral symmetry.

After implant placement, bilateral symmetry and appropriate three-dimensional projection were verified intraoperatively prior to wound closure. Figure 6A-6D illustrates the immediate postoperative appearance in one representative patient, demonstrating adequate gluteal contour, volume distribution, and symmetry following tension-free layered closure. Proper placement of bilateral Blake drains is also shown, reflecting the standard postoperative protocol used to minimize dead space and reduce the risk of fluid collection. The total bilateral operative time was less than 60 minutes in all cases.

Postoperative follow-up was conducted as part of routine clinical care. The first postoperative visit was scheduled at seven days after surgery, followed by weekly visits for the subsequent three weeks. Long-term follow-up was then continued, with a mean postoperative follow-up duration of approximately 24 months.

**Figure 7:**
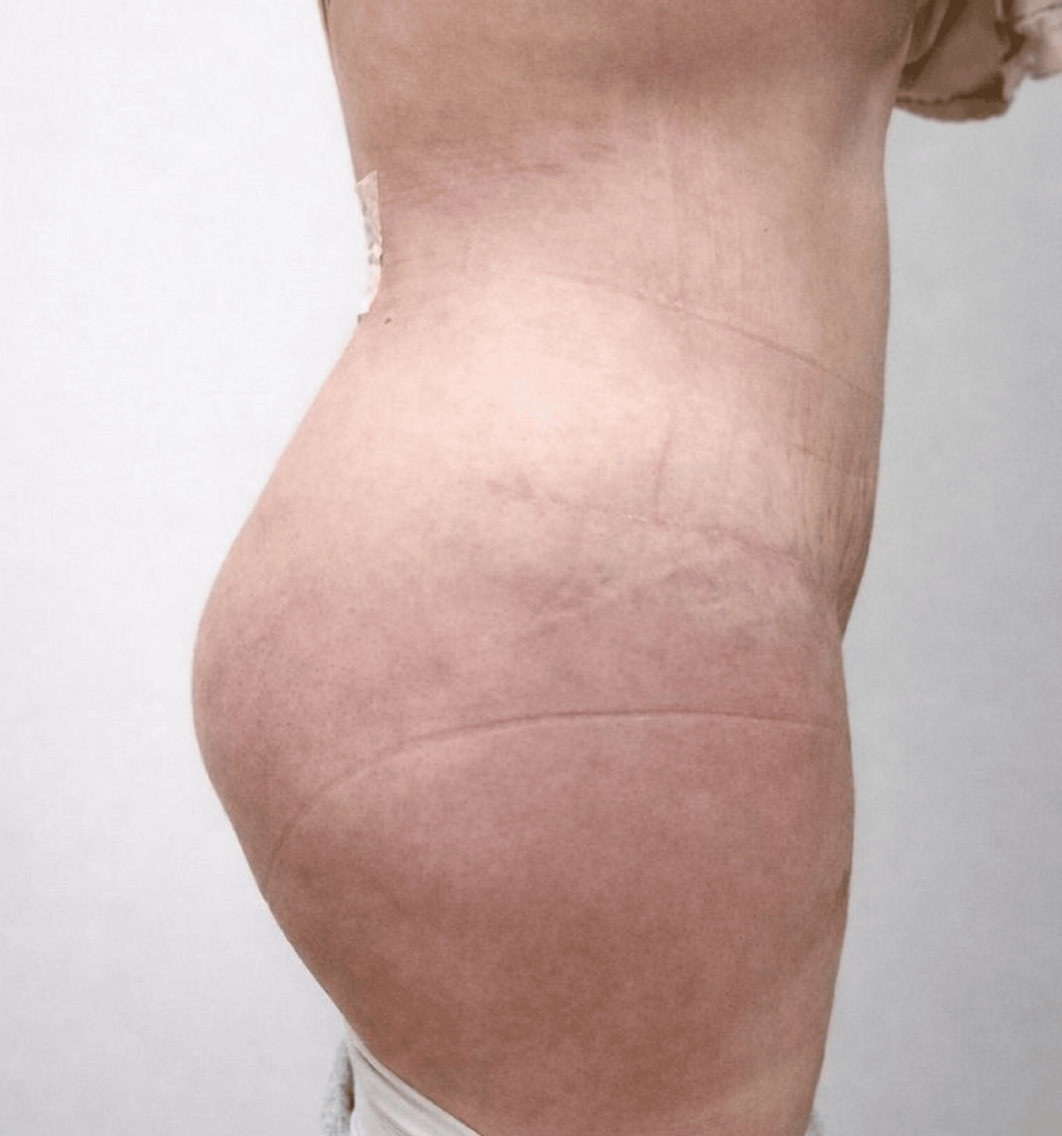
Early postoperative follow-up at one month Lateral view demonstrating maintained gluteal projection and contour at one month after surgery, with resolution of immediate postoperative changes and no evidence of wound-related complications.

Postoperative evaluation was conducted during routine clinical follow-up visits as part of standard postoperative care. Figure 7 depicts the clinical outcome at one month of follow-up, showing stable gluteal projection, preserved volume, and maintained bilateral symmetry without clinically evident complications.

Postoperative complications were actively assessed at each follow-up visit through clinical examination and targeted questioning, rather than passively reported. Specific attention was given to wound healing, seroma, hematoma, signs of infection, implant position, palpability, and neurologic symptoms. Functional outcomes, particularly pain or discomfort while sitting, were specifically and systematically assessed at every visit.

One patient developed a chronic seroma affecting a single gluteus approximately one year after surgery. This case required surgical reintervention, implant replacement, and placement of a drain for 21 days, with complete resolution. Additionally, one patient developed a postoperative hematoma at 15 days after surgery, which was managed by reopening the drain, resulting in complete resolution without further sequelae.

Intraoperative findings, postoperative complications, and overall clinical and aesthetic outcomes were systematically recorded. Given the descriptive nature of the study, data analysis was limited to descriptive statistics. Categorical variables were summarized as frequencies and percentages, and continuous variables were described using measures of central tendency, as appropriate. No inferential statistical analyses were performed.

## Results

A total of 17 female patients were included in the study. Baseline demographic and clinical characteristics are summarized in Table 1. The median age was 30 years (25-34), with a median height of 1.65 m (1.62-1.66) and a median body mass index of 28 kg/m² (27-29). None of the patients had documented medical comorbidities at the time of surgery. A history of previous aesthetic surgical procedures was present in all patients; 11 patients (64.7%) had undergone one prior aesthetic procedure, five patients (29.4%) had undergone two procedures, and one patient (5.9%) had undergone three procedures.

**Table 1:** Demographic, clinical, and operative characteristics of the study population (N=17) N=total number of patients. *Values are expressed as n (%). **Values are expressed as median (q25-q75).

Variable	Value
Sex (female)	17 (100%)*
Age (years)	30 (25-34)**
Height (m)	1.65 (1.62-1.66)**
Body mass index (kg/m²)	28 (27-29)**
Operative time (minutes)	60 (52-60)**
Comorbidities	0 (0%)*
Previous aesthetic surgeries: one	11 (64.7%)*
Previous aesthetic surgeries: two	5 (29.4%)*
Previous aesthetic surgeries: three	1 (5.9%)*
Implant type (round)	17 (100%)*
Implant volume (cc)	360 (360-415)**
Intramuscular implant placement	17 (100%)*
Postoperative complications: none	15 (88.2%)*
Postoperative complications: seroma	1 (5.9%)*
Postoperative complications: hematoma	1 (5.9%)*

All patients underwent bilateral intramuscular gluteal augmentation using round silicone implants placed entirely within the gluteus maximus muscle. Implant volumes ranged from 360 to 415 cc, with a median volume of 360 cc. The median total operative time for bilateral procedures was 60 minutes (52-60), reflecting a consistent operative duration across the cohort.

From an intraoperative and early aesthetic standpoint, adequate gluteal projection and bilateral symmetry were achieved in all cases, as confirmed prior to wound closure. No intraoperative difficulties related to implant positioning or pocket creation were documented. Tension-free layered closure was successfully performed in all patients, with bilateral placement of Blake drains according to the standardized postoperative protocol (Figure 6A-6D).

During postoperative follow-up, no cases of implant palpability, malposition, or herniation were observed. From a functional perspective, no patients reported chronic pain, discomfort while sitting, or functional limitations attributable to the procedure. Early postoperative clinical outcomes demonstrated stable contour and volume preservation, as illustrated at the one-month follow-up (Figure 7).

Regarding postoperative complications, 15 patients (88.2%) experienced an uneventful postoperative course. One patient (5.9%) developed a late seroma, and one patient (5.9%) presented with a postoperative hematoma. No cases of wound dehiscence, surgical site infection, implant rotation, or acute postoperative pain were documented during the follow-up period.

## Discussion

Gluteal augmentation with implants continues to represent a technically demanding procedure, largely due to the complex anatomy and dynamic function of the gluteus maximus muscle, as well as the heterogeneity of surgical approaches described in the literature. Although intramuscular placement has been widely adopted as a means of improving implant coverage and reducing palpability, published outcomes reveal substantial variability in complication rates and long-term stability. Large reviews and multicenter experiences, such as those reported by Sinno et al. [6], Oregi et al. [7], and Mofid et al. [8], underscore that intramuscular positioning alone does not guarantee uniform safety or predictability, particularly when differences in pocket design, incision choice, implant type, and adjunctive procedures are introduced.

In this context, the present case series demonstrates that a standardized intramuscular technique based on direct-vision dissection of the FROD space can be associated with a low complication rate and consistent aesthetic outcomes. In our cohort, 88.2% of patients experienced an uncomplicated postoperative course, with only isolated cases of seroma and hematoma and no instances of infection, implant malposition, sciatic nerve injury, or explantation. These results compare favorably with the higher overall complication rates reported in mixed-technique cohorts summarized by Sinno et al. and Oregi et al., where rates exceeding 20% have been documented. Importantly, those studies pooled data from heterogeneous surgical planes and composite augmentation strategies, limiting the ability to attribute outcomes to a single, well-defined technique [6,7].

The issue of postoperative infection deserves particular attention. Landfald reported a case of severe postoperative infection following intramuscular gluteal augmentation performed through an intergluteal fold incision, emphasizing the inherent contamination risk of this anatomical region and the potential for serious complications even when the implant is placed intramuscularly [9]. In contrast, no infectious complications were observed in the present series. While direct comparisons cannot be made from a single case report, this contrast highlights that the safety of intramuscular augmentation depends not only on the plane of dissection but also on meticulous pocket control, hemostasis, and incision selection. The absence of infection in our series suggests that controlled dissection under direct visualization and strict respect for anatomical boundaries may contribute to reducing this risk.

From a neuroanatomical standpoint, sciatic nerve injury represents one of the most feared complications of gluteal implant surgery. Kairinos and Jessop reported the first documented case of sciatic nerve transection following prosthetic gluteal augmentation, occurring during submuscular dissection and underscoring the vulnerability of the nerve when dissection extends into deep caudal planes with limited visualization [10]. Importantly, the authors emphasized that the intramuscular plane offers a relative protective advantage, as muscle fibers below the implant act as a barrier shielding the sciatic nerve. In the present series, no cases of sciatic nerve injury, transient sciatica, or postoperative neurologic deficits were observed. This finding supports the concept that controlled intramuscular dissection performed under direct visualization, with strict respect for anatomical landmarks and avoidance of caudal danger zones, renders the risk of sciatic nerve injury exceedingly low. Together, the absence of both infectious and neurologic complications reinforces the role of precise anatomical dissection and standardized technique in optimizing the safety profile of intramuscular gluteal augmentation.

Operative time represents another relevant point of comparison. The median operative time of approximately 60 minutes observed in this series is notably shorter than that reported by Trignano et al. [11], who described mean operative times of approximately 100 minutes for intramuscular augmentation performed under tumescent local anesthesia via an intergluteal approach, and by Aslani [12], whose large series of 423 patients undergoing dual-plane composite augmentation reported average operative times close to 97 minutes. Although these studies addressed different surgical objectives and included additional steps such as lipotransference or anesthetic infiltration, the contrast underscores how limiting dissection to a single, well-defined intramuscular plane and avoiding adjunctive procedures can improve operative efficiency. From a perioperative standpoint, shorter operative times may reduce anesthetic exposure and associated morbidity, an aspect that remains underexplored in aesthetic surgery literature.

From an aesthetic perspective, consistent gluteal projection and bilateral symmetry were achieved in all patients in the present series, with no cases requiring revision surgery. While formal patient-reported outcome measures were not employed, the absence of implant-related aesthetic complaints during follow-up suggests satisfactory clinical outcomes. The exclusive use of round implants may have contributed to this predictability. Notably, Aslani [12] reported a progressive shift away from anatomical implants in favor of round designs, citing improved reliability and fewer orientation-related complications, a trend that aligns with the implant selection strategy used in this study.

The relevance of optimizing primary intramuscular augmentation is further underscored by the work of Colli et al., who reported a series of secondary procedures addressing complications and failures following gluteal implant surgery [13]. Their analysis demonstrated that, despite initial placement within muscle-based planes, long-term issues such as muscle atrophy, implant migration, and palpability may develop and ultimately require complex revision surgery. These findings emphasize that the intramuscular plane alone does not ensure long-term safety or durability if technical execution and pocket control are suboptimal.

Complementary evidence regarding the role of extensive muscular coverage and technical standardization is provided by Petit et al., who described a retrospective series of 100 primary gluteal augmentations performed using a submuscular implant placement technique [14]. Although anatomically distinct from the intramuscular approach, their results demonstrated acceptable operative times and a favorable safety profile, with delayed wound healing and implant flipping being the most frequently reported complications. Importantly, the authors highlighted that complete muscular coverage contributed to implant protection, reduced palpability, and predictable aesthetic outcomes.

Taken together, these studies suggest that the long-term safety of implant-based gluteal augmentation depends less on the specific muscular plane selected and more on meticulous anatomical dissection, standardized pocket creation, and strict respect for muscular boundaries. In this context, the low early complication rate and stable implant positioning observed in the present intramuscular series support the clinical value of a controlled, direct-vision technique aimed at optimizing primary outcomes and potentially reducing the need for secondary interventions.

The reproducibility of this technique should be interpreted within the context of surgical experience. The described approach involves controlled intramuscular dissection under direct visualization and requires detailed anatomical knowledge of the gluteus maximus and surrounding danger zones. Therefore, the outcomes reported in this series may not be directly generalizable to surgeons early in their learning curve or to settings without adequate training and standardization. When performed by experienced surgeons within controlled clinical environments, this technique appears reproducible and may offer a predictable intramuscular implant placement.

It should be noted that some limitations of the present study exist. The relatively small sample size and retrospective design limit the ability to perform inferential statistical analyses or to identify predictors of complications or aesthetic outcomes. In addition, the absence of a comparative control group precludes direct conclusions regarding superiority over alternative implant planes or composite augmentation techniques. The lack of validated patient-reported outcome measures also restricts the objective quantification of patient satisfaction. These considerations are consistent with the descriptive nature of the study and should be interpreted within this methodological context.

Despite these considerations, the importance of systematically reporting and following patients treated with a standardized and anatomically controlled intramuscular technique should be emphasized. By focusing on a single surgical plane, a uniform implant type, and a reproducible operative protocol, this study reduces technical heterogeneity and allows a clearer assessment of procedural safety, operative efficiency, and early aesthetic consistency. The documentation of low complication rates, absence of implant-related morbidity, and stable aesthetic outcomes highlights the clinical relevance of reporting structured experiences that prioritize surgical precision and patient safety.

Future research should build upon these findings through prospective studies with longer follow-up, the incorporation of validated patient-reported outcome instruments, and comparative analyses with alternative or composite augmentation strategies. Within the current body of evidence, the present study supports controlled intramuscular gluteal augmentation with round implants as a safe and reproducible approach when performed using meticulous direct-vision anatomical dissection.

## Conclusions

This case series demonstrates that intramuscular gluteal augmentation performed using a controlled, direct-vision dissection of the FROD space assisted by macro-bipolar energy is a feasible and reliable surgical approach in carefully selected patients. By emphasizing anatomical precision, standardized pocket creation, and technical reproducibility, this technique offers a pragmatic refinement of conventional intramuscular augmentation rather than a conceptual departure from established principles. The findings of this study are limited to short- and mid-term outcomes observed within a controlled clinical setting and should be interpreted in the context of surgeon experience. Although limited by its descriptive design and sample size, the present experience supports the role of meticulous intramuscular technique optimization as a meaningful strategy to enhance safety, efficiency, and aesthetic consistency when performed by surgeons with comparable expertise. Further prospective studies are warranted to validate these findings and to better define the long-term performance of this approach within the broader spectrum of gluteal augmentation techniques.
